# Prevalence, knowledge and attitude toward electronic cigarette use among male health colleges students in Saudi Arabia—A cross-sectional study

**DOI:** 10.3389/fpubh.2022.827089

**Published:** 2022-10-05

**Authors:** Sary Alsanea, Ziyad Alrabiah, Sana Samreen, Wajid Syed, Rawan M. Bin Khunayn, Nasser M. Al-Arifi, Miteb Alenazi, Sultan Alghadeer, Abdulaziz Alhossan, Abdulrahman Alwhaibi, Mohamed N. Al-Arifi

**Affiliations:** ^1^Department of Pharmacology and Toxicology, College of Pharmacy, King Saud University, Riyadh, Saudi Arabia; ^2^Department of Clinical Pharmacy, College of Pharmacy, King Saud University, Riyadh, Saudi Arabia; ^3^Volunteer Researcher, Drug and Poison Information Center (DPIC), Department of Clinical Pharmacy, College of Pharmacy, King Saud University, Riyadh, Saudi Arabia; ^4^College of Medicine, Imam Mohammad Ibn Saud Islamic University, Riyadh, Saudi Arabia; ^5^College of Medicine, Almaarefa University, Riyadh, Saudi Arabia; ^6^Department of Clinical Pharmacy, King Saud University Medical City, Riyadh, Saudi Arabia

**Keywords:** smoking, electronic cigarettes (e-cigarettes), health care students, tobacco, Saudi Arabia

## Abstract

**Background:**

Health care professionals have an important role in increasing awareness about smoking harms and serving as role models. This study aims to assess knowledge, attitude and perception toward electronic cigarettes (ECs) as well as prevalence of ECs use among male health colleges students.

**Method:**

This is a cross-sectional survey-based study conducted among students in the male campus of five different health colleges over a 4-month period from February 2020 to May 2020. Descriptive analysis was used to assess the knowledge, perception and attitude, and inferential testing was used to evaluate the association of different participant's variables and knowledge toward ECs usage using SPSS.

**Results:**

A total of 333 students were included in the analysis. Most of students (*n* = 205; 61.6%) had never used ECs, while 22.8 and 15.6% used them for recreational and smoking cessation purposes, respectively. Focusing on ECs users from each college individually, medical students had the highest prevalence followed by dental, pharmacy and nursing students (47.4, 40.7, 34.5, and 32%, respectively). Many students had misconceptions and a low level of knowledge about ECs, such as recognizing them as smoking-cessation tools and not knowing whether toxic and carcinogenic components levels in ECs are similar to conventional cigarettes, respectively. Medical students had significantly higher knowledge compared to dental students [3 (2) vs. 2 (1); *p* = 0.033]. Moreover, smokers were less knowledgeable than non-smokers [2.5 (1) vs. 2.1 (1), *p* = 0.027]. At least 62.8% of students perceived using ECs as a fashionable alternative smoking method and 59.2% believed that they may become a gateway for smoking addiction. Only 120 (36.0%) health colleges students were confidently able to advise smokers regarding ECs.

**Conclusion:**

Our study highlights an increased trend of ECs use accompanied with insufficient knowledge and several misconceptions about ECs among health colleges students. This was associated with a negative influence on their attitude toward ECs use, which would potentially lead to negative consequences on public health.

## Introduction

An increasing trend among young individuals nowadays, particularly students, is the use of Electronic Cigarettes (ECs), which has been concerning, as it may potentially associate with worsening of the public health (1, 2). The national institute of drug abuse (NIDA) defines ECs as battery-operated devices, also known as vaping devices, or electronic nicotine delivery systems (ENDS), that allow users to inhale an aerosol, usually containing nicotine and flavorings, for recreational use (3). Thus, one of the justifications of using ECs is to help quit smoking, as they are generally perceived safer alternatives for conventional cigarettes. However, most of the population is unaware of the fact that they contain other chemicals and metals (4) and can deliver higher levels of nicotine than conventional cigarettes, which can elicit more addiction and predispose users to health issues, particularly cardiac problems (5). According to earlier reports, toxins contained in ECs, especially nicotine, are harmful to the human body particularly during adolescence, since the exposure to nicotine during that period of age can have long-lasting con-sequences, such as behavioral and neurological effects (6–8). Additionally, the use of ECs was shown to promote lung injury, as reported by the Center for Disease Control and Prevention (CDC). Moreover, many toxins delivered by ECs (3) or conventional cigarettes have been associated with a higher risk of different types of cancer, among which lung cancer is the most common one (7, 8).

Knowing the prevalence of EC use among young population—considered the major consumers of ECs—is imperative in order to combat this issue. In the United States, more than 1.78 million middle and high school students reported using ECs (7, 9). A recently published survey from the United States also revealed ECs as the most popular tobacco product used during adolescence (10). In addition, the prevalence of EC use between 2017 and 2019 increased significantly in teenagers from 11.7 to 27.5%, respectively (10). Focused on the current users among young adults in the United States, another study showed a significant hike, particularly in the former and never smokers, when compared to adults and elderly who exhibited no difference or gradually decrease when the prevalence was assessed in 2014 to 2018 (young adults: 5.1 vs.7.6%, *p* < 0.001) (11). In Britain, another study published on teenage students aged between 15 and 16 years showed a high prevalence (37.3%) (12). In Malaysia, it reached up to 86.5% and is more common among students aged ≥19 (13). In China, a recent study published in 2021 showed an increase in the prevalence of ECs use among teenage and young adults since 2015, especially population aged between 15 and 24 years (14). In Europe, the prevalence of EC use among young adults increased from 7.2 to 15% in 2017 (15, 16). In the Middle East, despite the wide range of prevalence, a recent study conducted on the general population in Jeddah, Saudi Arabia showed an increased rate of 51.4%, with the majority being high school and college students (17).

Health care students have an important role in improving public awareness toward health-related topics and behaviors, including smoking and using of ECs. However, to fulfill this purpose, the prevalence of ECs uses and the level of knowledge, perception and attitude toward ECs among this category must be initially assessed. A recent study conducted in 2020 on medical students in Riyadh assessing the prevalence of ECs use showed 12.2% (18). In 2019, Almutham and colleagues demonstrated that 10.6% of medical students from Qassim University have tried ECs at one time or another (19). In 2019, another study conducted on health sciences colleges in Jeddah reported ~28% of students using ECs, which was higher by 14% than conventional cigarette smokers (~14%) (20). With respect to the prevalence of ECs users in different health colleges in Saudi Arabia, it has been estimated to reach 7.9, 13.4, and 29% among dental, pharmacy, and medical students, respectively (20, 21). In general, a battery of studies from both developed and developing countries including Saudi Arabia reported the widespread use of ECs among college students (7, 12, 18–20, 22, 23). However, despite the aforementioned few studies that investigated the use of ECs among health colleges students in Saudi Arabia, little is known about students' knowledge and attitude toward ECs. In other words, given their crucial role as future health care professionals, their knowledge, attitude and behavior regarding the use of ECs is important, as they will contribute into promoting healthy habits and counseling patients about smoking cessation (24, 25). Thus, this study aimed to assess and compare knowledge, perception and attitude toward ECs as well as the prevalence of ECs use among health colleges students.

## Methods

### Study design, sample and data collection

A cross-sectional, paper-based survey study was conducted between February 2020 and May 2020 among male students enrolled in various health colleges at King Saud University, including the College of Medicine, Pharmacy, Dentistry, Nursing and Applied Medical Sciences. Students aged ≥18 years who expressed a willingness to complete the survey were included. At the beginning of the survey and following the study title, a disclosing statement followed by consenting & agreement to use filled information for publication purposes was highlighted. Students below the age of 18 and students from other disciplines were excluded from the study.

### Sample size estimation

According to previous reports, the prevalence of ECs used among health colleges students in Saudi Arabia was 27.7% (20). Given that, the sample size was calculated using the following equation: *n* = z^2^ × p × q/d^2^ where n is the minimum sample size, z is the constant (1.96), p is the prevalence of ECs (among health care students, it was 0.277), q is (1 – p), Z is the standard normal deviation of 1.96 corresponding to the 95% confidence interval and d is the desired degree of accuracy. *n* = (1.96)^2^ × 0.277 (1–0.277)/(0.05)^2^ = 308.

### Questionnaire design

Questionnaire used in this study was developed after a review of available literature pertaining to the knowledge, prevalence and attitude about EC use among health colleges students (26–32). It consisted of 17 questions, divided into 2 sections. The first section included 6 questions dedicated toward obtaining information about the student's health colleges, year of study, gender, smoking status, history of use of ECs, and whether being taught smoking cessation topics. The second section comprised of 11 questions about knowledge, perceptions and attitude regarding ECs use, with a total of 5, 3, and 3 questions, respectively. Knowledge-related questions were either three-choice-based or Likert scale-based questions. For three-choice-based questions, one answer was considered correct, and a score of one was given if it was chosen. For the Likert scale-based question, two answers were considered right (strongly agree and agree were considered the same; and disagree and strongly disagree were also considered the same), in which the participant would be given a score of one if anyone of them was chosen. The overall knowledge score for each student ranged between 0 and 5.

To ensure readability of the questionnaires and ease of administration, a pilot study was conducted among randomly selected small sample of pharmacy students (*n* = 10). The result of the pilot study was not included in the final analysis. The reliability of the questionnaire was calculated using Cronbach's Alpha value of (0.70), indicating that questionnaire was reliable to carry out the study.

### Statistical analysis

A descriptive analysis was conducted to assess the prevalence and sociodemographic factors of the study population. Chi-square test was used for categorical variables analysis whenever applied. Kruskal Wallis and Mann-Whitney tests were used for continuous variables analysis whenever applied. Repeated Mann-Whitney with Benferroni's adjustment method was utilized to determine the difference between health colleges. The simple linear regression analysis was performed to find out any correlation between students' variables and knowledge toward ECs. The data was analyzed using Statistical Package for Social Sciences version 26.0 (SPSS Inc., Chicago, IL, USA), and *p* value of <0.05 was considered statistically significant.

## Results

**Figure 1:**
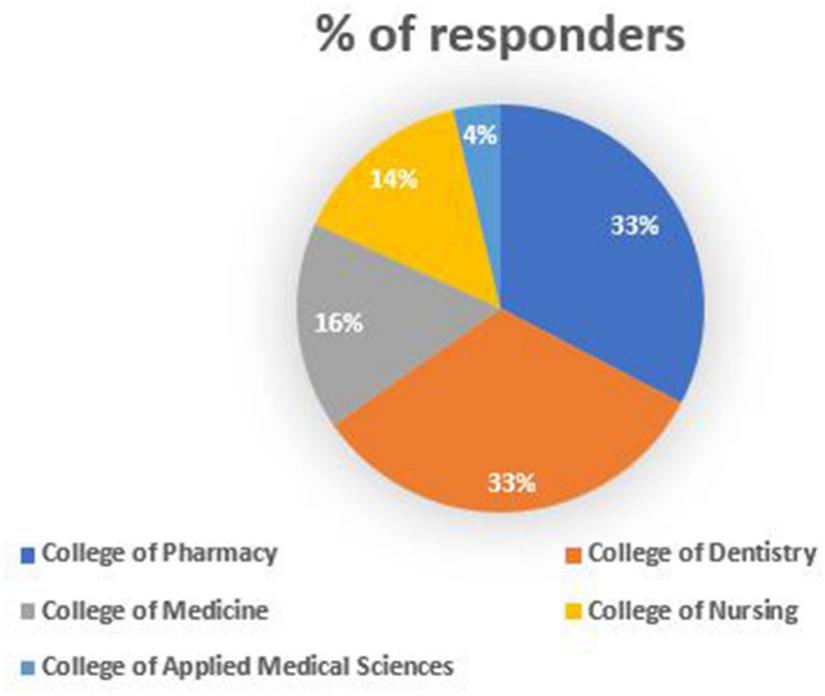
Proportion of responders from each health colleges.

A total of 400 questionnaires were distributed on health colleges students, among which three hundred and forty-six responded, resulting in a response rate of 86.5%. Proportion of responders from each health colleges is provided in Figure 1. Given the lowest response from applied medical sciences students, they were excluded from the study, leaving 333 students subjected to analysis related to the prevalence of use, knowledge, perception and attitude toward ECs.

Our analysis revealed a total of 159 (47.7%) of students had been or were smokers at the time of the study being conducted. Taking each college separately, smoking behavior (including all those who were ex-smokers and current smokers) was more prevalent among dental students, followed by nursing, medical and pharmacy students (58.4, 56.0, 43.9, and 35.4%, respectively). When students were asked about using ECs, 38.4% confirmed using them, while 61.6% denied it. However, when we compared the prevalence of smoking (47.7%) with the prevalence of ECs use (38.4%), the higher prevalence of smoking in general indicates that some smokers were probably smoking conventional cigarettes or using smoking devices other than ECs like “traditional or electronic hookah” with/out ECs. Further investigation on ECs showed that they were used either for recreational (59.4%) or smoking cessation purposes (40.6%). The higher percentage of those using them recreationally resonates with students being more lenient to considering ECs as smoking devices. When EC users among health colleges were compared, taking each college's students separately, students enrolled in medical college represented the highest, followed by dental, pharmacy and nursing colleges (47.4, 40.7, 34.5, and 32%, respectively). Focusing only on the ECs users in each health college, when students were compared on the basis of recreational use, nursing students represented the highest (62.5%), while others were comparable (~59.0%). Additionally, when the same approach was used with respect to smoking cessation purposes, pharmacy, dental and medical students were comparable (~41%), whereas nursing students had the lowest proportion of 37.5%. More details can be found in Table 1.

**Table 1:** Prevalence of smoking and use of e-cigarettes among the students of health care colleges (*n* = 333).

**Variables**	**Pharmacy *n* (%)**	**Dentistry *n* (%)**	**Medicine *n* (%)**	**Nursing *n* (%)**	**All colleges *n* (%)**
**Smoking status**					
Smoker	27 (23.9)	38 (33.6)	10 (17.54)	17 (34.0)	92 (27.6)
Ex-smoker	13 (11.5)	28 (24.8)	15 (26.32)	11 (22.0)	67 (20.1)
Non-smoker	73 (64.6)	47 (41.6)	32 (56.14)	22 (44.0)	174 (52.3)
**Use of e-cigarettes**					
No	74 (65.4)	67 (59.3)	30 (52.6)	34 (68.0)	205 (61.56)
Yes, for recreational purpose	23 (20.4)	27 (23.9)	16 (28.1)	10 (20.0)	76 (22.82)
Yes, for smoking-quitting purpose	16 (14.2)	19 (16.8)	11 (19.3)	06 (12.0)	52 (15.62)

Regarding the students' knowledge about ECs, two-thirds [214 (41.4 and 22.8%)] failed to recognize ECs as non-smoking cessation products. In addition, more than the half [181 (31.8 and 22.5%)] had incorrectly believed that ECs are safe and did not know that they have adverse effects like conventional cigarettes. Furthermore, at least 208 (62.5%) had wrong perceptions and lacked the knowledge about the harmful components contained in and delivered by ECs compared to conventional cigarettes. Further information regarding this knowledge is provided in Table 2.

**Table 2:** Health care students' knowledge toward e-cigarettes.

**Statement**	**Yes *n* (%)**	**No *n* (%)**	**I don't know *n* (%)**
ECs are well-recognized smoking cessation products^*^.	138 (41.44)	118 (35.44)	76 (22.82)
ECs do not have adverse effects.	106 (31.83)	152 (45.65)	75 (22.52)
ECs produce vaporized nicotine only.	120 (36.03)	125 (37.54)	88 (26.43)
Carcinogens present in ECs are similar to those produced by conventional cigarettes^*^.	139 (41.74)	91 (27.33)	102 (30.63)
Longer puffing period with ECs increases the dose of inhaled carcinogens and cytotoxic agents.	178 (53.45)	45 (13.51)	110 (33.03)

* Missing data.

Despite the previously noticed variation in the proportion of EC users between health colleges, there was no difference in the level of knowledge between students, except for medical students, who had a significantly higher levels compared to dental students only [Median (IQR): 3 (2) vs. 2 (1); *p* = 0.033]. Further details about scores are provided in Table 3. Students' categorization based on the smoking status indicated that non-smokers were significantly more knowledgeable compared to smokers [2.5 (1) vs. 2(1); *p* = 0.027], as shown in Table 4. Additionally, regression analysis was performed to find any relation between knowledge toward ECs and students variables. Covering smoking cessation topic previously had a significantly positive impact with on the knowledge score (B = 0.404; SE = 0.127; *p* = 0.002).

**Table 3:** Median score of knowledge toward e-cigarettes according to health colleges.

**Groups**	**Median (IQR)**	***P*** **-value**
College of dentistry^#^	2 (1)	0.033
College of medicine^#^	3 (2)	
College of nursing	2 (1)	
College of pharmacy^*^	3 (1)	

* Kruskal-Wallis test;

# *p*-value < 0.05 using repeated Mann-Whitney with Benferroni's adjustment.

**Table 4:** Median score of knowledge toward e-cigarettes according to smoking status.

**Groups**	**Median (IQR)**	***P*** **-value^#^**
Smoker- Ex-smoker	2 (1)	0.027
Non-smoker	2.5 (1)	

# Mann-whitney test.

With respect to the perception and attitude of students toward ECs, more than half [197 (59.2%)] agreed that ECs may become a gateway to smoking among non-smokers and could lead to smoking addiction in the conventional cigarette smokers, as shown in Table 5. In addition, approximately two-thirds [209 (62.8%)] believed that ECs are used as a fashionable alternative to conventional cigarettes than a method to quit smoking. Surprisingly, although not shown in Table 5, when all health colleges students were asked about their perception on the most knowledgeable, appropriate health care provider that should educate public about the risk of ECs and lead smoking cessation programs, the majority ranked respiratory therapist (37.2%) the first (despite its exclusion from our analysis as it is a subspecialty in the applied medical sciences college), followed by physician (26.4%), pharmacist (15%), dentist (10.5%) and nurse (10.5%). Lastly, the attitude of students toward EC smoking was greatly influenced by their knowledge and perception, where only 34.2% didn't support using ECs as smoking cessation method and only around 26.7% were willing to ask patients about using ECs, despite their insufficient knowledge about this topic. This was negatively and substantially associated with a decline in their confidence on discussing this topic with smokers. Further information is provided in Table 5.

**Table 5:** Perception and attitude of health care students toward e-cigarettes.

**Statement**	**Agree *n* (%)**	**Disagree *n* (%)**	**I do not know *n* (%)**
• ECs may become a gateway for smoking in non-smokers and smoking addiction in conventional tobacco cigarette smokers.	197 (59.2)	49 (14.7)	87 (26.1)
• ECs are used as fashionable alternative of conventional cigarettes more than smoking cessation method.	209 (62.8)	47 (14.1)	77 (23.1)
• I support using ECs as a smoking cessation method for those who want to quit smoking.	154 (46.2)	114 (34.2)	65 (19.5)
• If I don't have enough knowledge about e-cigarettes, I shouldn't ask a patient whether they used them or not.	179 (53.8)	89 (26.7)	65 (19.5)
• I can confidently advise smokers about ECs.^*^	120 (36.0)	137 (41.1)	72 (21.6)

* Missing data.

## Discussion

Our study assessed the knowledge, attitude and perception toward ECs as well as the prevalence of ECs use among health colleges students at King Saud University. In general, the prevalence of smoking was found to be (47.7%), which is higher than the previously reported rates by Sychareun and colleagues (5.1%) (33), by Nasser and colleagues (12.4%) (34) and by Amin and colleagues (13.7%) (35). Despite the variation between countries, our findings suggest that smoking behavior among Saudi health colleges students is more prevalent compared to those from different countries.

Regarding the prevalence of ECs users among our students with reference to each college separately, medical students represented the highest (47.4%), followed by dental, pharmacy and nursing participants −40.7, 34.5, and 32%, respectively. This increase in the user rate among medical students is higher than locally and globally reported rates from Al-Faisal University and Qassim University in Saudi Arabia (12.2 and 10.6%, respectively) (18, 19) and University of Minnesota (15.3%) (36). Similarly, the proportion of ECs users among pharmacy students was higher compared to those at Midwestern University in the United States, 34.5 vs. 14%, respectively (37). Regarding nursing students, although Canzan and colleagues showed an increased percentage of nurses using ECs (25), the proportion in our nursing students is approximately reduced by half (57.2 vs. 32%). Overall, our study, beside the current literature, confirmed the popularity of ECs use among health colleges students in developed and developing countries (18, 19, 25, 29, 33, 35) attributed to their beliefs toward ECs use as either recreational or as a smoking cessation product and the amount of knowledge about ECs that they have gained throughout their education.

Regarding the students' knowledge about ECs, our study surprisingly revealed a reduced level of knowledge among health colleges students. For example, only 35.44% of the participants knew that ECs were not considered smoking cessation products and only 45.65% agreed that ECs had adverse effects. In addition, 36.03% stated that ECs produce vaporized nicotine only, which contradicts the reality that they contain potentially carcinogenic and non-carcinogenic toxins beside nicotine (36). It is worth mentioning that smoke from ECs contains far fewer carcinogenic particles than conventional cigarettes smoke (37). However, 41.7% of our students incorrectly believed that cancer-causing agents found in ECs are similar to those existing in conventional cigarettes, and 30.6% didn't know about the carcinogenic components of ECs smoke. Interestingly, the low level of knowledge about ECs seems to be common among health colleges students reflected by reports of Habib et al. and Guckert et al., where 69.4% of medical students and 81.6% of dental students believed ECs were less harmful compared to conventional cigarettes, respectively (18, 38). Although the level of knowledge is clearly low among our participants, 53.45% correctly reported longer EC puffing increases the amount of inhaled toxins and carcinogens.

According to the students' perception toward ECs, 62.8% confirmed that ECs are used as a fashionable alternative of conventional cigarettes, and 59% agreed on ECs being potentially a gateway to smoking for non-smokers and smoking addiction for conventional cigarette smokers, which goes hand in hand with the previous report by Alzahrani et al. on medical students where 50.6% agreed that ECs were addictive (39). Similarly, Almutham and colleagues showed that 49.6% of medical students believed ECs were addictive (19). Overall, it is clear that conventional cigarette use has been replaced by EC, with no scientific basis. Consequently, public health authorities are deeply concerned about the increasing popularity of ECs, and the WHO and FDA have voiced concerns about their safety and the effects of nicotine on the body. This becomes more concerning when it comes to the reality that many youths and young adults use ECs exclusively, which would potentially predispose them to smoking and nicotine addiction on the long term. Lastly, although the data is not shown, 26.6% of health colleges students (after excluding those who selected respiratory therapist (35.8%) believed that physicians were the most knowledgeable health care professionals to provide advice and counseling to EC users and establish and manage smoking cessation programs). Despite that, knowledge scores show no differences between students except for medical vs. dental students. Thus, all health colleges students could provide advice regarding ECs. Nevertheless, since smoking cessation is an important topic incorporated in the pharmacy therapeutic courses and the fact that pharmacists are more accessible to the public than other health care professionals, they are more appropriate to discuss the use of ECs and smoking cessation methods and to run smoking cessation programs than other health care professionals (40).

With respect to the health colleges students' attitude toward ECs, 46.2% supported using them as a smoking cessation product without any scientific basis. This reiterates the results re-ported by Alzahrani et al., where one-third of medical students agreed on using ECs as a smoking cessation method (39). Despite that, a study from China reported that only 9.5% EC users had successfully quit smoking (14). Cumulatively and not surprisingly, only 36% of our participants felt confident in discussing ECs with patients.

The current study has some limitations. First, the results were based on a self-completed questionnaire, which might increase the possibility of social desirability bias or recall bias. Second, the results were derived from a single university in Saudi Arabia, making them not-representative of others and not generalizable globally. Third, the study did not involve female students as it was conducted in the male campus of university, given the easier access to male students found while spreading the questionnaire. In spite of these limitations, our study suggests more emphasis on increasing the awareness of health colleges students toward ECs and correcting the misconceptions regarding using them as a smoking cessation method to make them more competent in raising public awareness on ECs.

## Conclusion

Our study highlights an increasing trend of ECs use with low levels of knowledge among health colleges students living in the capital of Saudi Arabia, Riyadh. More importantly, nursing students had less familiarity with ECs knowledge, which could have a harmful impact on their health. In addition to the nicotine in the ECs, there were certain additional dangerous compounds that, when inhaled, directly reach the lungs and have serious negative effects. Therefore, we advocate the implementation of programs or workshops that educate students about the misconceptions surrounding ECs as smoking cessation tools and their consequences on overall health. An Inter-professional approach to education that addresses the safety and utility of using ECs could be the most practical way to address such a complex issue.

## Data availability statement

The raw data supporting the conclusions of this article will be made available by the authors, without undue reservation.

## Ethics statement

The studies involving human participants were reviewed and approved by Institutional Review Board (IRB) approval was obtained from King Saud University College of Medicine with the following reference number (IRB-E-21-6370). The patients/participants provided their written informed consent to participate in this study.

## Author contributions

AAlw, MA-A, ZA, and SAls: conceptualization and methodology. SS and WS: software, validation, and formal analysis. AAlw, MA-A, ZA, SAls, MA, SAlg, AAlh, RB, and NA-A: investigation. SAls and WS: data curation. AAlw, SS, MA-A, ZA, and SAls: writing–original draft preparation. AAlw and MA-A: supervision. AAlw: project administration. All authors reviewed the manuscript. All authors contributed to the article and approved the submitted version.

## Funding

This project was funded by Researchers Supporting Project (Project Number RSP-2021/81), King Saud University, Saudi Arabia.

## Conflict of interest

The authors declare that the research was conducted in the absence of any commercial or financial relationships that could be construed as a potential conflict of interest.

## Publisher's note

All claims expressed in this article are solely those of the authors and do not necessarily represent those of their affiliated organizations, or those of the publisher, the editors and the reviewers. Any product that may be evaluated in this article, or claim that may be made by its manufacturer, is not guaranteed or endorsed by the publisher.
