# Supplementary material for: Prevalence, knowledge and attitude toward electronic cigarette use among male health colleges students in Saudi Arabia—A cross-sectional study
Source: Front Public Health. 2022 Oct 5;10:827089. doi: 10.3389/fpubh.2022.827089 (PMC9580394; doi:10.3389/fpubh.2022.827089)
Supplement: Supplementary file 1 [file Table_1.doc]

**Assessment of Prevalence, Knowledge and Attitude towards Electronic Cigarette Use among Male Health Colleges Students in Saudi Arabia—A Cross-sectional Study**

**Disclosure:** Participation in this study is voluntary and none of the questions can be used to identify your identity. Completing this survey indicates your consenting & agreement to use this information for publication purposes.

**Our study aims to:**

1. Evaluate the health colleges’ students’ perception and knowledge about e-cigarettes and
2. Evaluate the benefits/efficacy and dangers associated with their utilization as a smoking-quitting and social/recreational tools, respectively

**Questions:**

1. **What is your college?**
   1. College of Applied Medical Sciences
   2. College of Dentistry
   3. College of Medicine
   4. College of Nursing
   5. College of Pharmacy
2. **What year are you classified into?**
   1. First year
   2. Second year
   3. Third year
   4. Fourth year
   5. Fifth year
   6. Sixth year
3. **Have you ever been taught smoking-cessation topic at your college? If so, which year?**
   1. Never
   2. First year
   3. Second year
   4. Third year
   5. Fourth year
   6. Fifth year
   7. Sixth year
4. **What is your gender?**
   1. Male
   2. Female
5. **Please select your smoking habit:**
   1. Smoker
   2. Ex-smoker
   3. Non-smoker
6. **Have you ever used e-cigarettes? Is so; was it for recreational or smoking-quitting purpose?**
   1. No
   2. Yes, for recreational purpose
   3. Yes, for smoking-quitting purpose
7. **E-cigarettes are well-recognized smoking-cessation products (i.e. help in reducing and quitting smoking.)**
   1. Yes
   2. No
   3. I do not know
8. **E-cigarettes do not have adverse effects (like cough, difficulty in breathing and eye irritation) compared to conventional tobacco cigarettes.**
   1. Yes
   2. No
   3. I do not know
9. **E-cigarettes produce vaporized nicotine only.**
   1. Yes
   2. No
   3. I do not know
10. **I would support using e-cigarettes as a smoking-cessation method for those who want to quit smoking.**
    1. Strongly agree
    2. Agree
    3. I do not know
    4. Disagree
    5. Strongly disagree
11. **Although combustion (burning) of tobacco is absent in e-cigarettes, there are some potential carcinogens present in e-cigarettes similar to those produced by conventional tobacco cigarettes.**
    1. Yes
    2. No
    3. I do not know
12. **Longer puffing period with e-cigarettes increases the dose of inhaled carcinogens and cytotoxic agents.**
    1. Strongly agree
    2. Agree
    3. I do not know
    4. Disagree
    5. Strongly disagree
13. **E-cigarettes may become a gateway for smoking in non-smokers and smoking addiction in conventional tobacco cigarettes smokers.**
    1. Strongly agree
    2. Agree
    3. I do not know
    4. Disagree
    5. Strongly disagree
14. **E-cigarettes are used as fashionable alternative of conventional cigarettes more than smoking-cessation method.**
    1. Strongly agree
    2. Agree
    3. I do not know
    4. Disagree
    5. Strongly disagree
15. **If I don’t have enough knowledge about e-cigarettes, I shouldn’t ask a patient whether they used them or not.**
16. Strongly agree
17. Agree
18. I do not know
19. Disagree
20. Strongly disagree
21. **I can confidently advise smokers about e-cigarettes.**
22. Yes
23. No
24. I do not know
25. **In your opinion, who should be the most knowledgeable health professional that should educate and counsel e-cigarettes users and lead smoking-cessation programs?**
26. Dentists
27. Nurse
28. Pharmacist
29. Physician
30. Respiratory Therapist

**References used for developing the survey:**

1. Effects of electronic cigarette smoking on human health. Eur Rev Med Pharmacol Sci . 2014;18(21):3315-9.
2. Assessing the carcinogenic potential of E-cigarette. Oral Oncol . 2018 Jun;81:111.
3. Electronic Cigarettes: Impact on Lung Function and Fractional Exhaled Nitric Oxide Among Healthy Adults. Am J Mens Health . Jan-Feb 2019;13(1):1557988318806073
4. Experimentation and correlates of electronic nicotine delivery system (electronic cigarettes) among university students – A cross sectional study. Saudi Dent J . 2016 Apr;28(2):91-5.
5. Cardiovascular Effects of Exposure to Cigarette Smoke and Electronic Cigarettes. J Am Coll Cardiol . 2015 Sep 22;66(12):1378-91.
6. Mining data on usage of electronic nicotine delivery systems (ENDS) from YouTube videos. Tob Control . 2013 Mar;22(2):103-6.
7. https://www.cdc.gov/tobacco/data_statistics/sgr/2020-smoking-cessation/fact-sheets/adult-smoking-cessation-e-cigarettes-use/index.html#:~:text=Some%20research%20suggests%20that%20using,cessation%20than%20less%20frequent%20use.
